# Targeting mitochondrial phosphatidylethanolamine alters mitochondrial metabolism and proliferation in hepatocellular carcinoma

**DOI:** 10.21203/rs.3.rs-7042684/v1

**Published:** 2025-07-15

**Authors:** Tim Heden, Melina Mancini, Cameron McCall, Robert Noland, Wagner Dontas

**Keywords:** Phospholipids, phosphatidylethanolamine, mitochondria, liver cancer, metabolism

## Abstract

Mitochondrial metabolism is crucial for hepatocellular carcinoma (HCC) to thrive. Although phospholipids modulate mitochondrial metabolism, their impact on metabolism in HCC remains unknown. Here we report that the mitochondrial phospholipidome is unaltered in HCC mitochondria, suggesting HCC maintain their mitochondrial phospholipidome to enable efficient metabolism and promote thriftiness. Consistent with this, silencing phosphatidylserine decarboxylase (PISD), the inner mitochondrial membrane protein that generates mitochondrial phosphatidylethanolamine (PE), in HEPA1-6 cells impairs mitochondrial metabolism of fatty acid and glucose-derived substrates and reduces electron transport chain I and IV abundance. Moreover, PISD deficiency increased mitochondrial superoxide generation and altered mitochondria dynamics by augmenting mitochondrial fission, mitophagy, and mitochondrial extracellular efflux. Despite compensatory increases in anaerobic glycolysis and peroxisome fat oxidation, mitochondrial PE deficiency reduced DNA synthesis and cell proliferation, effects associated with reduced mTOR signaling and peptide levels. We conclude that targeting mitochondrial PE synthesis may be a viable therapy to slow HCC progression.

## Introduction

The most common form of liver cancer is hepatocellular carcinoma (HCC), which accounts for approximately 90% of liver cancer cases (1). Current therapies for HCC have limited effectiveness, while the development of more effective treatment options has been slow due to a lack of understanding of its molecular pathogenesis and progression. However, emerging evidence in the field has identified that most cancers, including HCC, display altered phospholipid metabolism (2–4). Phospholipids are fundamental for the proper structural integrity of cell membranes (5). Rapidly growing tumors upregulate phospholipid synthesis to match the tumor growth rate with the need for new cell membranes, while lipid synthesis also alters membrane properties to protect cancer cells from endogenous and exogenous insults (6). Mitochondrial metabolism is crucial for HCC growth and survival (7–10) whereas mitochondrial phospholipids are fundamental for the structural integrity of mitochondrial membranes (5) and through lipid-protein interactions modulate mitochondria protein localization, stability, and function (11). Although the whole cell phospholipidome is altered in HCC (12–14), it is not clear whether mitochondrial phospholipid content is altered in HCC.

Phosphatidylethanolamine (PE) is one of the most abundant phospholipids within mammalian cells and is primarily synthesized through the CDP-ethanolamine pathway (Kennedy pathway) or within mitochondria through decarboxylation of phosphatidylserine (PS) to PE via the mitochondrial enzyme phosphatidylserine decarboxylase (PISD) (15–18). The PISD pathway generates most mitochondria PE in normal cells, but its importance in the context of HCC is undefined. A previous study demonstrated in Chinese Hamster Ovary cells that silencing PISD results in severe mitochondrial defects and reduces the cell growth rate (19), suggesting that PISD may be a promising target for slowing cancer cell progression. However, another study reported that reducing PISD in skeletal muscle did not alter mitochondrial respiration but increased DNA synthesis (20), a finding that might promote tumor progression. In breast cancer, overexpression of PISD reduced tumor initiating potential and metastasis (21, 22). We next searched The Human Protein Atlas (23) (www.proteinatlas.org) which revealed PISD was a potential prognostic marker in melanoma and pancreatic cancer, but not HCC. In melanoma, low expression of PISD was more favorable for survival, while in pancreatic cancer high expression of PISD was more favorable for survival, suggesting that PISD may have a different role depending on the cancer type. Taken together, PISD deficiency may alter mitochondrial bioenergetics and cell proliferation differentially depending on cancer type, while the role of mitochondrial PE in HCC metabolism remains unknown.

The purpose of this study was to characterize the phospholipidome of mitochondria isolated from a mouse model of chemical induced HCC and to examine how silencing PISD and reducing mitochondria PE in liver cancer cells alters metabolism, mitochondrial function, and cell proliferation. Our data indicates that the phospholipidome of liver tumor mitochondria is unaltered compared to mitochondria from adjacent non-tumor liver tissue, indicating that the mitochondrial phospholipidome remains intact in HCC and is likely essential to maintain mitochondria function. Consistent with this, silencing PISD impaired mitochondria bioenergetics, an effect associated with reduced DNA synthesis rates and cell proliferation.

## Results

### Mitochondrial lipidomics in a mouse model of HCC

**Figure 1:**
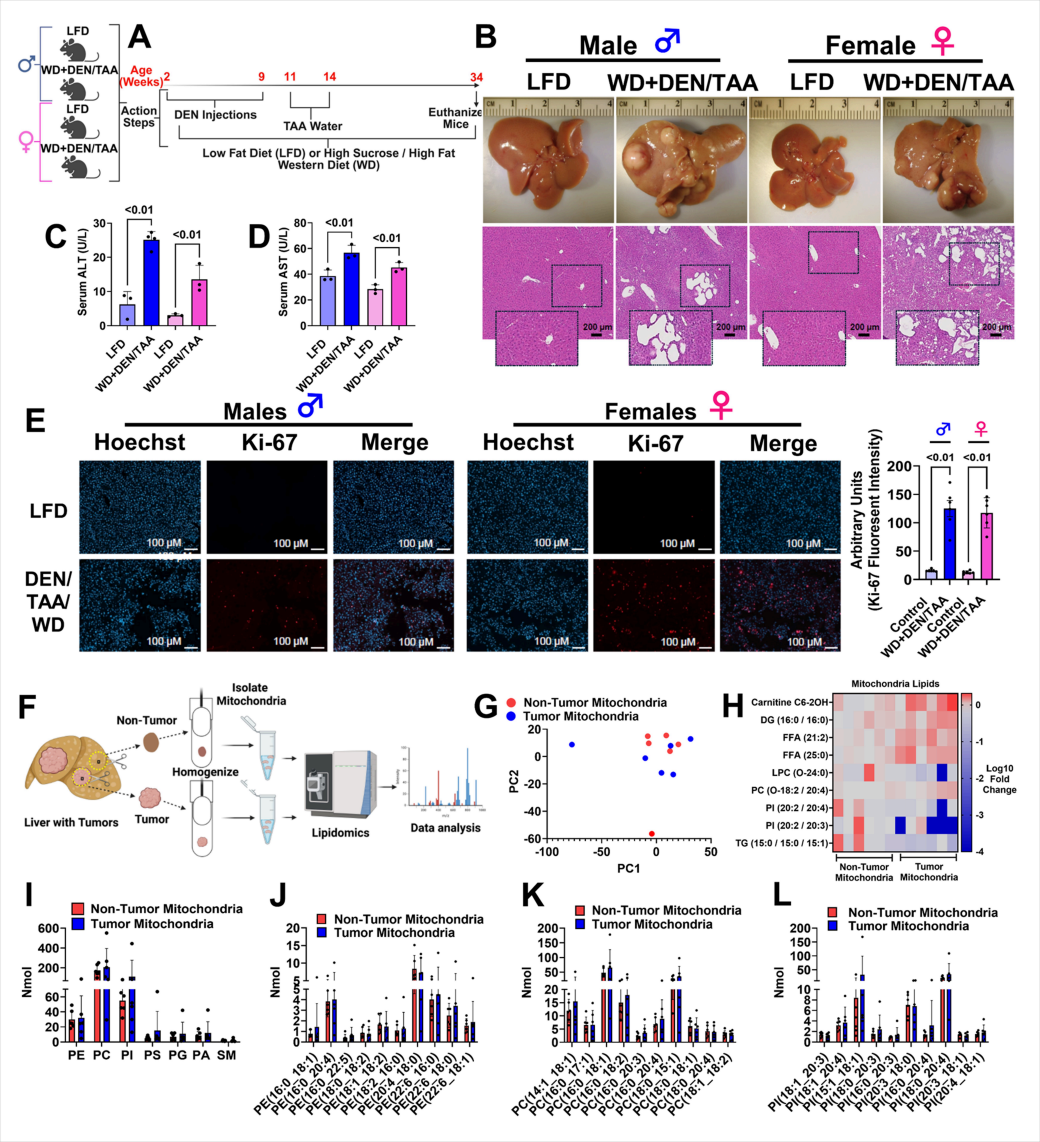
The mitochondrial phospholipidome is unaltered in hepatocellular carcinoma (HCC). **A** Schematic showing overall mouse study design and protocol to induce HCC using diethylnitrosamine (DEN) and thioacetamide (TAA). **B** Images of representative liver dissected from the mice (top) with a corresponding hematoxylin and eosin (H&E) stain (bottom). **C** Serum ALT activity. **D** Serum AST activity. **E** Nuclei (Hoechst) and Ki-67 (proliferation marker) fluorescent images of liver sections. **F** Schematic showing workflow for mitochondrial isolation from non-tumor and tumor tissue and subsequent lipidomics analysis. **G**PCA plot of mitochondrial lipids between non-tumor and tumor mitochondria. **H**Heatmap of select lipids that were differentially altered between non-tumor and tumor mitochondria. **I** Abundance of mitochondrial phospholipids in non-tumor and tumor mitochondria. **J** Abundance of select phosphatidylethanolamine species in non-tumor and tumor mitochondria. **K**Abundance of select phosphatidylcholine species in non-tumor and tumor mitochondria. **L** Abundance of selected phosphatidylinositol species in non-tumor and tumor mitochondria. The data are presented as S.D.

To determine whether the mitochondrial phospholipidome is altered in HCC, we induced HCC in wildtype C57BL/6J male and female mice using a modification of a previously published protocol involving the carcinogens diethylnitrosamine (DEN) and thioacetamide (TAA) in addition to a high sucrose / high fat Western diet (WD) (24) (Fig. 1A). Our protocol was effective at inducing large visible liver tumors and HCC by 34 weeks of age in both male and female mice (Fig. 1B). Hematoxylin and eosin staining revealed HCC with clusters of dense nuclei indicating a high rate of cell proliferation (Fig. 1B). Serum alanine transaminase (ALT) and aspartate aminotransferase (AST), markers of liver damage that are elevated in HCC, were increased in the WD + DEN/TAA treated groups (Fig. 1C–1D). Moreover, the fluorescent intensity of the proliferation marker Ki-67 was elevated in liver sections of both male and female mice in the WD + DEN/TAA group (Fig. 1E), further confirming the development of HCC in our model.

Mitochondria from non-tumor liver tissue and liver tumor tissue were isolated and rapidly frozen in liquid nitrogen until being prepped for quantitative lipidomic analysis (Fig. 1F). For the lipidomic analysis, samples from both male and female mice were used, including three males and three females, and each mouse had an adjacent non-tumor mitochondria and tumor mitochondria fraction analyzed. A total of 1631 mitochondria lipid species were detected (**Supplementary Table 1**). A PCA plot did not reveal significant separation in lipid species between non-tumor and tumor mitochondria (Fig. 1G), indicating that the mitochondria from liver tumors are largely unaltered compared to non-tumor mitochondria.

However, nine total lipids were significantly different between non-tumor and tumor mitochondria (Fig. 1H). This included an increased tumor mitochondria abundance of carnitine C6-2OH, diacylglycerol 16:0 / 16:0, free fatty acids 21:2 and 25:0, and PC O-18:2 / 20:4, while the lipid species lysophosphatidylcholine O-24:0, PI 20:2 / 20:4 and 20:2 / 20:3, and TG 15:0 / 15:0 / 15:1 were significantly reduced in tumor mitochondria (Fig. 1H). The total abundance of the phospholipid species PE, phosphatidylcholine (PC), phosphatidylinositol (PI), phosphatidylserine (PS), phosphatidylglycerol (PG), phosphatidic acid (PA), and sphingomyelin (SM) was not different between non-tumor and tumor mitochondria (Fig. 1I–1L), suggesting that the phospholipidome of tumor mitochondria is intact and required to maintain proper mitochondrial function and support rapid cell proliferation.

### Silencing PISD reduces mitochondrial PE content and alters mitochondrial bioenergetics

**Figure 2:**
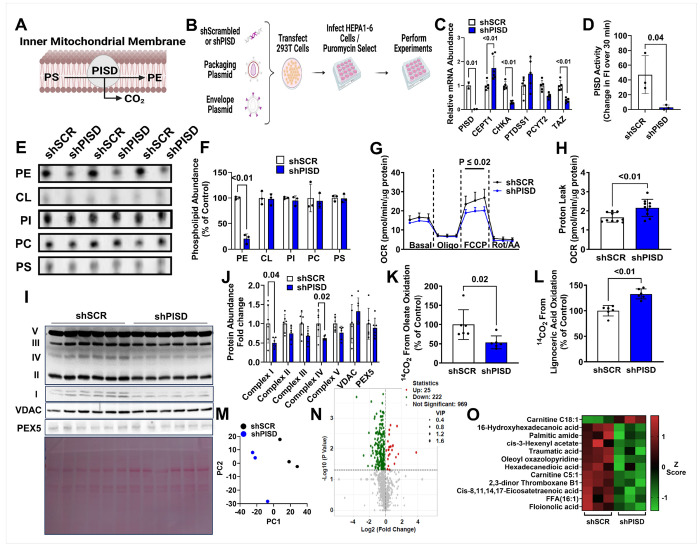
Silencing PISD in HEPA1-6 cells reduces mitochondrial lipid metabolism and electron transport chain Complex I and IV abundance. **A** Schematic illustrating PISD enzyme reaction with phosphatidylserine (PS) to form CO_2_ and phosphatidylethanolamine (PE). **B** Schematic showing generation of shScrambled (shSCR) and shPISD lentivirus in 293T cells and subsequent infection and gene silencing in HEPA1-6 cells. **C** mRNA abundance of various genes involved in phospholipid metabolism. **D** PISD activity. **E-F** Thin layer chromatography images (**E**) and densitometry quantification (**F**). G Seahorse mitochondrial stress test. **H** Proton leak, calculated by subtracting oxygen consumption rates (OCR) during basal conditions from OCR during oligomycin (oligo) treated conditions. **I-J** Western blot image (**I**) and densitometry quantification (**J**). **K** 1-^14^C oleate oxidation. **L** 1-^14^C lignoceric acid oxidation. **M** PCA plot of untargeted metabolomics data. **N** Volcano plot of untargeted metabolomics data. **O** Heatmap of select lipid metabolism related metabolites that were differentially expressed between shSCR and shPISD treated cells. The data are presented as S.D.

Given that the mitochondrial phospholipid content was similar between tumor and non-tumor mitochondria, it indicates that maintenance of mitochondrial phospholipid content may be important in HCC to promote optimal mitochondrial metabolism. To test this hypothesis, we sought to determine if mitochondrial phospholipid synthesis is a metabolic vulnerability that could be targeted to disrupt mitochondrial metabolism and impair HCC progression. We focused on PE synthesis, which in mitochondria is generated through a decarboxylation reaction of phosphatidylserine via the enzyme PISD (Fig. 2A), as a previous study has shown PISD deficiency reduces cell viability (19). To determine how inhibiting mitochondrial PE synthesis impacts HCC metabolism, a 2nd generation lentivirus system was used to knockdown PISD (Fig. 2B). A control scrambled (shSCR) lentivirus or shPISD lentivirus were made using HEK293T cells, and subsequently the lentiviruses produced were used to infect HEPA1-6 cells (Fig. 2B). The shPISD lentivirus was effective as PISD mRNA abundance was significantly reduced (Fig. 2C) as well as PISD activity (Fig. 2D). We also determined if shPISD treated cells had altered levels of other genes involved in phospholipid metabolism, and the mRNA abundance of choline / ethanolamine- phosphotransferase-1 (CEPT1) was increased in shPISD treated cells, while the mRNA abundance of choline kinase alpha (CHKA) and tafazzin (TAZ) displayed modest, but significant, reductions (Fig. 2C). We next isolated mitochondria and measured the phospholipid content of the major mitochondrial phospholipids including PE, cardiolipin (CL), PI, PC, and PS using thin-layer chromatography (TLC). Mitochondrial PE was significantly reduced in shPISD treated cells, while no significant differences between shSCR and shPISD treated cells were observed in other mitochondrial phospholipids (Fig. 2E–F).

To determine how loss of mitochondrial PE impacted mitochondrial bioenergetics and metabolism, we performed a series of cell phenotyping experiments. A Seahorse mitochondrial stress test revealed that shPISD treated cells had reduced FCCP-stimulated maximal aerobic capacity, although basal respiration rates were not altered (Fig. 2G). Mitochondrial proton leak increased in shPISD treated cells (Fig. 2H) suggesting that the membranes of PE deficient mitochondria leak more protons into the mitochondrial matrix independently of ATP synthase (i.e. ATP production). The reduced capacity for oxidative metabolism was associated with reduced abundance of Complex I and IV of the electron transport chain, located on the inner mitochondrial membrane (Fig. 2I–J). These data suggest that mitochondrial PE may play a crucial role in maintaining the stability of these complexes. The protein abundance of the voltage-dependent anion channel (VDAC), which is located on the outer mitochondrial membrane and functions to transport mitochondrial metabolites, was not altered in shPISD treated cells suggesting that PE synthesis mainly impacts inner mitochondrial membrane proteins. The peroxisome marker PEX5 protein abundance was not altered (Fig. 2I–J), indicating that peroxisome abundance was not altered. Complete oxidation of 1-^14^C oleate into ^14^CO_2_ was reduced in shPISD treated cells, indicating a reduction in mitochondrial fatty acid oxidation (Fig. 2K). This coincided with an increase in the complete oxidation of 1-^14^C lignoceric acid into ^14^CO_2_, which is a very long chain fatty acid that is initially metabolized in peroxisomes, suggesting that peroxisomal fatty acid metabolism increases to compensate for mitochondrial dysfunction and reductions in fatty acid metabolism in mitochondria (Fig. 2L). This increase in peroxisomal lignoceric acid oxidation was due to increased flux of very long chain fatty acids through peroxisomal fatty acid catabolism, not increased peroxisomal abundance, and greater capacity for very long chain fatty acid metabolism.

To better gauge alterations in global cell metabolism induced by mitochondrial PE deficiency, a metabolomics analysis on whole cell samples was performed (**Supplemental Table 2.**). A PCA plot revealed a clear separation between shSCR and shPISD treated cells (Fig. 2M). A volcano plot revealed a total of 1 489 metabolites that were identified (Fig. 2N), with a differential expression analysis revealing that 247 of these metabolites were differentially expressed between shSCR and shPISD treated cells, with 222 metabolites lower in abundance and 25 higher in abundance in shPISD treated cells. A fatty acid metabolite that was more abundant in shPISD treated cells included carnitine C18:1, which is a metabolite required for the transfer of long chain fatty acids across the inner mitochondrial membrane for oxidation. These data suggest that carnitine C18:1 might accumulate due to reduced flux through fatty acid oxidation. Fatty acid species that were less abundant in shPISD treated cells included 16-hydroxyhexadecanoic acid, palmitic amide, cis-3-hexenyl acetate, traumatic acid, oleoyl oxazolopyridine, hexadecanedioic acid, carnitine C5:1, 2,3-dinor thromboxane B1, Cis-8,11,14,17-eicosatetraenoic acid, FFA(16:1), and floionolic acid (Fig. 2O).

### PISD deficiency promotes anaerobic glycolysis

**Figure 3:**
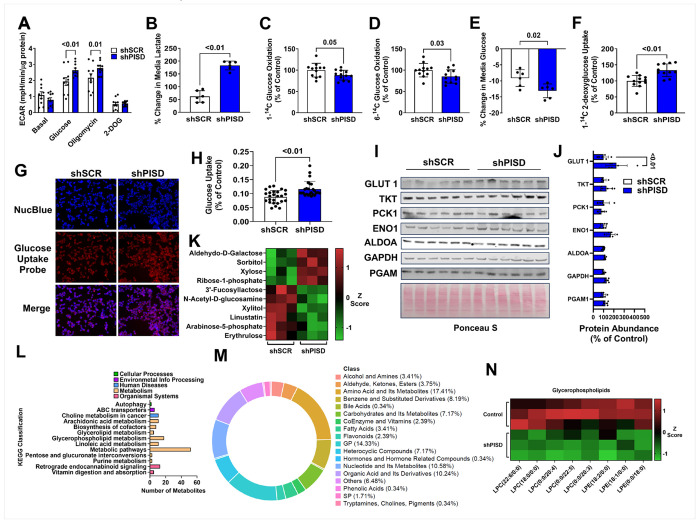
Enhanced anaerobic glycolysis but reduced aerobic glycolysis in PISD silenced HEPA1-6 cells. **A** Extracellular acidification rates (ECAR) during a seahorse mitochondrial stress test. **B** Percentage change in media lactate over a 24-hour period. **C** 1-^14^C glucose oxidation. **D** 6-^14^C glucose oxidation. **E** Percentage change in media glucose concentration. **F** 1-^14^C 2-deoxyglucose uptake. **G-H** Representative fluorescent images (**G**) and quantification of fluorescent intensity (**H**). **I-J** Western blot images (**I**) and densitometry quantification (**J**). **K** Heatmap of carbohydrate related metabolites that were significantly different between shSCR and shPISD treated cells. **L** Number of metabolites in each KEGG classification that were significantly different between shSCR and shPISD treated cells. **M** Percentage of significantly different metabolites within each class. **N** Heatmap of significantly different glycerophospholipids. The data are presented as S.D.

Given that mitochondrial PE deficiency reduced mitochondrial fatty acid oxidation, we next tested if glycolysis was altered as a compensatory mechanism. A glycolysis stress test revealed that the extracellular acidification rate (ECAR), a measure of glycolysis, was not different between control and shPISD treated cells when glucose was absent from the media, indicating no change in endogenous glycogen metabolism (Fig. 3A). When glucose was subsequently added to the media, ECAR increased in both control and shPISD treated cells, but this increase was greater in shPISD treated cells (Fig. 3A). The shPISD treated cells also had elevated rates of lactate appearance in the media (Fig. 3B), indicating increased anaerobic glycolysis. A limitation of the glycolysis stress test is that the pH of the extracellular media is used, which is not only impacted by lactate but also by CO_2_. To specifically measure glucose oxidation and aerobic glycolysis, 1-^14^C glucose and 6-^14^C glucose were used. The 1 labeled carbon on glucose can enter either glycolysis or the pentose phosphate pathway, while the 6 labeled carbon on glucose only enters glycolysis (25). Both 1-^14^C and 6-^14^C glucose oxidation were significantly reduced in shPISD treated cells (Fig. 3C–3D), indicating both reduced aerobic glycolysis and flux through the oxidative branch of the pentose phosphate pathway. Glucose uptake, as assessed by the % decrease in glucose from the media over 24 h (Fig. 3E), 1-^14^C 2-deoxy-glucose uptake (Fig. 3F), or uptake of a fluorescent glucose analog (Fig. 3G–3H) all revealed a modest increase in glucose uptake in shPISD treated cells, which helps support increased anaerobic glycolysis. Consistent with increased glucose uptake in shPISD treated cells, GLUT 1 protein abundance increased (Fig. 3I–3J). Neither the protein abundance of the pentose phosphate pathway enzyme transketolase or enzymes involved in glycolysis including phosphoenolpyruvate carboxykinase 1 (PCK1), enolase, glyceraldehyde-3-phosphate dehydrogenase (GAPDH), or phosphoglycerate mutase (PGAM) were altered (Fig. 3I–3J), suggesting that increased anaerobic glycolysis with mitochondrial PE deficiency occurs due to increased glycolytic flux rather than alterations in protein machinery. Metabolomics also revealed alterations in several carbohydrate metabolites including increased abundance of aldehyde-d-galactose, sorbitol, and xylose with reduced abundance of 3’-fucosyllactose, N-Acetyl-D-glucosamine, xylitol, linustatin, arabinose-5-phosphate, and erythrulose in shPISD treated cells (Fig. 3K). Ribose-1-phosphate is a secondary metabolite in the pentose phosphate pathway that can be converted to ribose-5-phosphate, which is used for nucleotide synthesis. Thus, its accumulation in shPISD treated cells may indicate a reduced flux through the pentose phosphate pathway, which is consistent with the reduction in 1-^14^C glucose oxidation.

Consistent with our mitochondrial and glycolysis functional experiments, a Kyoto Encyclopedia of Genes and Genomes (KEGG) pathway analysis of the significantly impacted cell metabolites revealed that metabolic pathways were most impacted in shPISD treated cells (Fig. 3L). Additionally, the KEGG pathway analysis and a class count ring (Fig. 3M) indicated that glycerophospholipids were significantly impacted, with shPISD treated cells having lower abundance of the lysophosphatidylcholine (LPC) species LPC(22:6/0:0), LPC(18:0/0:0), LPC(0:0)/20:4), LPC(0:0)/22:5), and LPC(0:0)/20:3) as well as the lysophosphatidylethanolamine (LPE) species LPE(18:2/0:0), LPE(18:1/0:0), and LPE(0:0/18:0) (Fig. 3N).

### PISD deficiency increases mitochondrial superoxide production and alters mitochondrial dynamics

**Figure 4:**
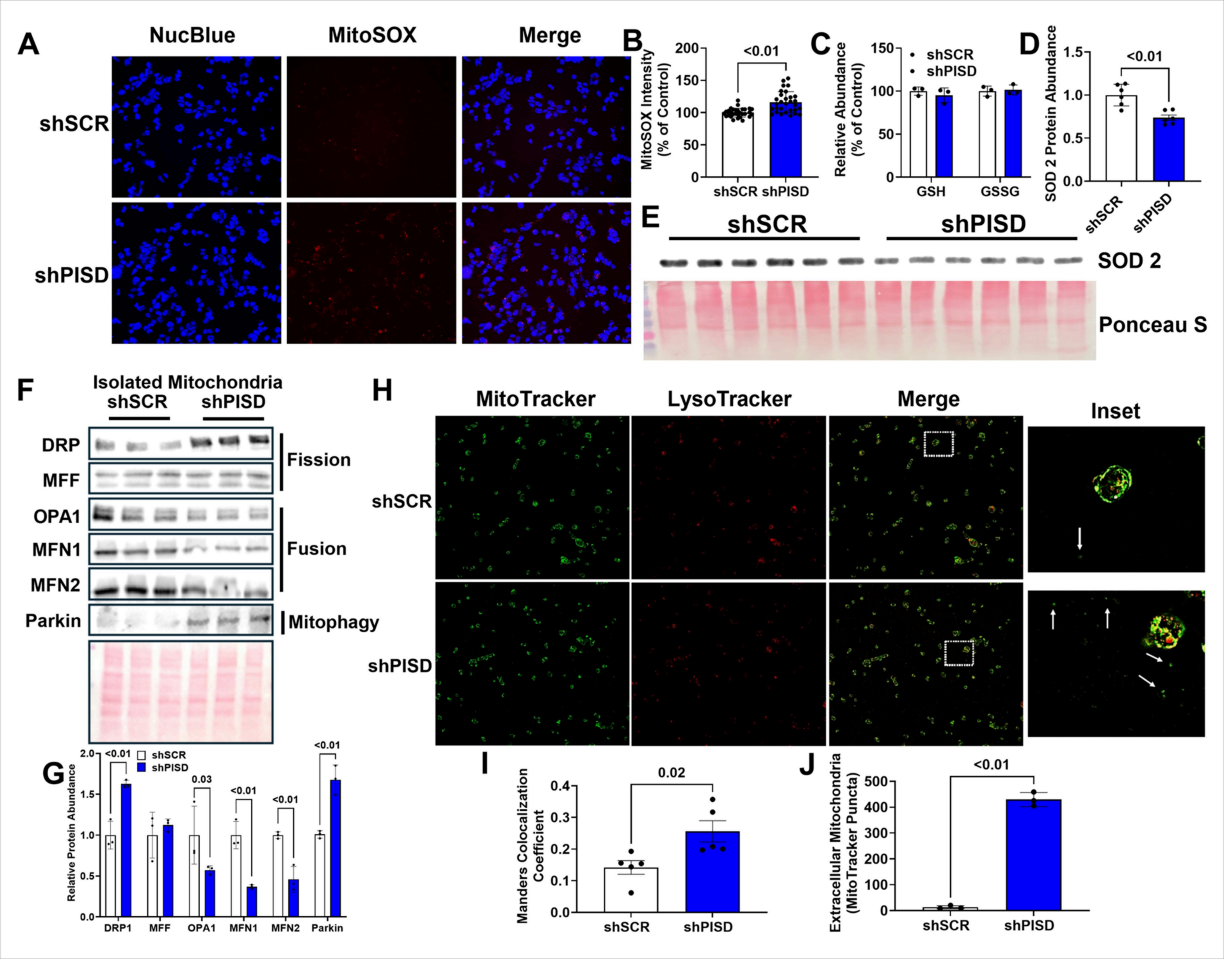
Silencing PISD increases mitochondrial superoxide production, mitochondrial fission, and mitophagy. **A** Representative images of MitoSOX staining. **B**Quantification of MitoSOX fluorescent intensity from images in A. **C**Relative abundance of reduced glutathione (GSH) and oxidized glutathione or glutathione disulfide (GSSG). **D** Abundance of superoxide dismutase 2 (SOD2). **E** Western blot image of SOD2 protein abundance. **F** Western blot image showing abundance of mitochondrial fission, fusion, or mitophagy related proteins. **G**Densitometry quantification of images shown in **F-H** Representative fluorescent images of MitoTracker and LysoTracker colocalization. **I**Manders colocalization coefficient of MitoTracker and LysoTracker. **J** Number of extracellular mitochondria puncta. The data are presented as S.D.

A cellular process that occurs during normal cellular metabolism is the generation of reactive oxygen species (ROS). Mitochondria generate the ROS species superoxide during electron transfer. Given that mitochondria are an important source of ROS, it was next tested whether mitochondrial PE deficiency altered mitochondria ROS production. The mitochondria specific superoxide indicator MitoSOX ^™^ (M36008, Thermo Fisher Scientific, Plaquemine, LA, USA) was used to assess mitochondrial superoxide production and revealed that shPISD treated cells had greater mitochondrial superoxide production, indicated by higher red fluorescence staining (Fig. 4A–4B), showing an increase in mitochondrial ROS production with mitochondrial PE deficiency. Cells have antioxidant defense enzymes in place to combat ROS. The primary antioxidant within cells is glutathione (GSH), and metabolomics revealed that PISD deficiency did not alter either GSH or its reduced form, GSSG, indicating that loss of GSH is not the cause of increased ROS production (Fig. 4C). GSH is localized to the cytosol, while superoxide dismutase 2 (SOD2) is localized to the mitochondrial matrix where it neutralizes superoxide originating from the respiratory chain. Therefore, we next measured the protein abundance of SOD2, which revealed shPISD treated cells had reduced protein abundance of SOD2 (Fig. 4D–4E). This suggests that PE deficiency disrupts the capacity of mitochondria to neutralize superoxide by reducing SOD2 protein abundance.

Previous studies have demonstrated that loss of PISD results in altered mitochondrial morphology which may impact mitochondria dynamics and the ability of mitochondria to undergo fusion, fission, and mitophagy. Furthermore, the excessive generation of ROS by mitochondria serves as a signal that targets mitochondria for mitophagy. Therefore, it was next determined if mitochondrial PE deficiency would alter mitochondrial dynamics. The protein abundance of dynamin-related protein 1 (DRP1), a marker of mitochondrial fission, was elevated in isolated mitochondria from shPISD treated cells (Fig. 4F–4G) suggesting mitochondrial PE deficiency increases fission, although mitochondrial fission factor (MFF) was not altered in shPISD treated cells (Fig. 4F–4G). The protein abundance of markers of mitochondrial fusion including optic atrophy protein 1 (OPA1), mitofusin 1 (MFN1), and mitofusin 2 (MFN2) were reduced in shPISD treated cells, which is consistent with an increase in mitochondrial fission and suggests that mitochondrial from shPISD treated cells undergo fission into smaller mitochondrial and oppose mitochondrial fusion into larger mitochondria (Fig. 4F–4G). Further, the protein abundance of the mitophagy marker Parkin was elevated in isolated mitochondria from shPISD treated cells (Fig. 4F–4G), indicating that loss of mitochondrial PE promotes mitophagy. To further assess mitophagy, the co-localization of mitotracker and lysotracker were used (Fig. 4H). shPISD treated cells had a significantly higher Manders colocalization coefficient suggesting greater mitophagy (Fig. 4I), which is consistent with an increase in the mitophagy marker Parkin. Unexpectedly, we also observed a rise in extracellular mitochondria around shPISD treated cells (Fig. 4H and 4J), suggesting that in addition to mitophagy, shPISD treated cells may get rid of dysfunctional mitochondria by releasing them extracellularly.

### PISD deficiency alters cell proliferation and protein homeostasis

**Figure 5:**
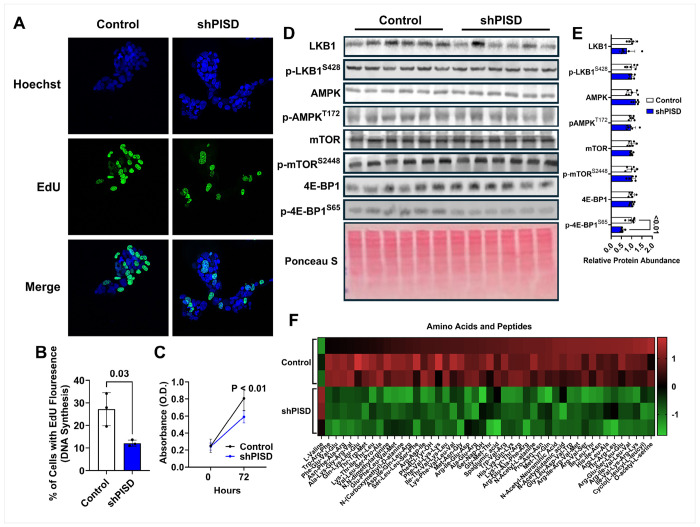
Silencing PISD reduces cell proliferation and disrupts amino acid homeostasis and mTOR signaling. **A** Representative fluorescent images of EdU staining. **B** Quantification of EdU fluorescence. **C** Absorbance of WST8 compound at baseline and 72 h. **D-E** Western blot images (**D**) and densitometry quantification (**E**). **F** Heatmap significantly affected amino acids and peptides in shSCR and shPISD cells. The data are presented as S.D.

Energetic stress and excessive ROS can slow cellular growth. Therefore, we next tested whether the alterations in metabolism in shPISD treated cells would alter DNA synthesis and cell proliferation. The fluorescent thymidine analog EdU was used to measure DNA synthesis during a one-hour period in full growth media (10% FBS). Fluorescence microscopy revealed that shPISD cells had a significantly lower number of cells with EdU fluorescence (Fig. 5A–B). Cell proliferation was also measured using the tetrazolium salt, WST-8, at baseline and 72 h later, and the absorbance produced at 72 h was significantly reduced in shPISD treated cells, suggesting reduced cell proliferation (Fig. 5C). To better understand mechanistically why shPISD cells reduced DNA synthesis and cell proliferation rates, the protein abundance of the known tumor suppressor liver kinase B1 (LKB1) and its downstream target and known energy sensor adenosine monophosphate-activated protein kinase (AMPK) were measured. The shPISD treated cells had normal protein abundance of liver kinase B1 (LKB1) as well as the protein abundance of phosphorylated LKB1 at Serine 428 (p-LKB1^S428^) (Fig. 5D–5E). Similarly, shPISD cells had a similar protein abundance of total and phosphorylated adenosine monophosphate activated protein kinase (AMPK) at Threonine 172 (p-AMPK^T172^) (Fig. 5D–5E), which is a downstream target of LKB1. Although the protein abundance of mechanistic target of rapamycin (mTOR) or its phosphorylation at serine 2448 was not different, the phosphorylation of mTOR’s downstream target eukaryotic translation initiations factor 4E-binding protein 1 (4E-BP1) at serine 65 was reduced. The 4E-BP1 protein binds to eukaryotic translation initiation factor 4E (eIF4E) to inhibit cap dependent transcription. Its affinity to bind to eIF4E depends on its phosphorylation status, with phosphorylation at serine 65 reducing its ability to bind eIF4E and inhibit cap dependent transcription. Therefore, reduced phosphorylation of 4E-BP1 in shPISD treated cells indicates reduced cap dependent translation, which is consistent with reduced cell growth.

Amino acids and peptides alter mTOR signaling. Our metabolomics analysis revealed that amino acids and related metabolites represented the largest class of affected metabolites accounting for 17.41% of the total (Fig. 5F). Valine was the lone amino acid in higher abundance in shPISD treated cells. Meanwhile, numerous other amino acid related metabolites including various tripeptides, tetrapeptides, and pentapeptides were all lower in abundance in shPISD treated cells (Fig. 5F), which may reduce mTOR signaling.

## Discussion

Although Otto Warburg initially hypothesized that the increase in glycolysis in cancer cells was due to reduced mitochondrial function (i.e. Warburg effect), subsequent studies have shown that proper functioning mitochondria are required for some cancer cells to thrive (26, 27). Mitochondria programming is heterogeneous in cancer and is highly adaptable to meet energy supply and demand within the ever-changing tumor microenvironment (28). The data herein further supports the notion that properly functioning mitochondria are essential for tumor cells to thrive. We find that isolated mitochondria from mouse HCC tumors maintain their phospholipidome compared to mitochondria isolated from adjacent, non-tumor mitochondria, indicating that normal mitochondrial phospholipid content might be required for HCC cells to thrive. Indeed, this idea was supported by experiments in HEPA1-6 cells that showed that reducing the expression of mitochondria PISD reduces mitochondria PE content, mitochondria respiration, and aerobic glycolysis. Furthermore, mitochondrial PE deficiency increased mitochondrial superoxide abundance, fission, mitophagy, and mitochondrial extracellular efflux, effects that were associated with reduced cell proliferation rates and mTOR signaling. Together, these data suggest that mitochondrial PE is required for the proper function of mitochondria in HCC cells and may be a vulnerability that could be targeted to slow HCC progression.

The transmembrane electron transport chain enzymes are embedded within the inner mitochondrial membrane and are tightly associated with membrane phospholipids. This tight association allows the coordinated assembly of specifically configured supercomplexes which allow electron transfer to function correctly. Our data suggests that PE is not only essential for optimal mitochondrial electron transport chain respiration but also the stability of electron transport chain complexes I and IV, but not complexes II, III, or V, in liver cancer. Consistent with our findings, other work has shown that mitochondrial PE is bound to bovine heart mitochondrial complex I and the catalytic activity of the enzyme is determined, in part, by the amount of PE present (29). In the porcine heart, PE interacts with complex I, particularly with various segments of the NDUFA11 subunit (30). In the bovine heart, mitochondrial PE is tightly bound to Complex IV (31). In Chinese hamster ovary cells, PISD deficiency led to reduced abundance of supercomplexes, which impaired electron transfer and mitochondrial respiration (19). Consistent with this, the impairments we observed in electron transport chain function were associated with reduced respiration rates and the complete oxidation of fatty acids and glucose, which mitochondria are required for. Thus, mitochondrial PE deficiency renders HCC cells less metabolically flexible, which may translate into being less robust and more vulnerable to treatment.

Mitochondria are the primary site of ROS production in cells (32). A total of 11 sites between Complex I – III of the electron transport chain within mitochondria produce ROS (superoxide or hydrogen peroxide) from substrate oxidation (32). A major mitochondrial antioxidant is SOD2 which catalyzes the conversion of superoxide radicals into oxygen and hydrogen peroxide (33). ROS production is normal and acts as an important second messenger in various intracellular signaling pathways (34). Excessive ROS can be detrimental to cells as they can interact and damage cell components and may play a role in the initiation of HCC (35, 36). However, ROS may be a double-edged sword, as after HCC is initiated excessive ROS in cancer cells can halt progression and trigger apoptosis in some contexts (36). Consistent with this, the slower cell proliferation observed herein with mitochondrial PE deficiency was associated with increased mitochondrial superoxide content and reduced SOD2 protein abundance. This cellular ROS stress may in turn play a role in slowing cell proliferation.

Mitochondria are highly dynamic organelles that are constantly undergoing fission, fusion, mitophagy, or transport cycles (37). These processes help maintain optimal mitochondrial and cellular function while also dictating mitochondrial morphology, number, quality, and cellular location. Previous reports have indicated that mitochondrial dynamics are altered in HCC. For instance, HCC mitochondria were reported to be shorter compared to mitochondria from adjacent, non-tumor tissue while increased mitochondrial fission promoted HCC cell survival (38). Consistent with this, MFN2 overexpression, which promoted mitochondrial fusion, reduced HCC cell proliferation (39). Mitophagy is also required for HCC cells to thrive, while mitophagy inhibition reduces HCC cell growth and promotes apoptosis (40, 41). Thus, increased mitochondrial fission and mitophagy in our model were likely an adaptation to promote HCC cell survival in the setting of mitochondrial PE deficiency, which reduced mitochondrial substrate metabolism and increased mitochondrial ROS generation. Unexpectedly, we also observed increased extracellular mitochondria around cells with mitochondrial PE deficiency, suggesting an increase in the extracellular release of dysfunctional mitochondrial to help maintain the quality of the mitochondrial pool. Cells can release mitochondria or parts of mitochondria in two fashions including 1) a non-membrane bound free form via secretory vesicles, similar to how hormones and neurotransmitters are released, or 2) through membrane-enclosed extracellular vesicles or EV’s, which can be referred to as migrasomes and in a process referred to as mitocytosis where mitochondria are positioned at the cell periphery or tip of protrusions, where they are then pinched off from the cell (42–46). To our knowledge, this is the first study to show that HCC cells efflux dysfunctional mitochondria.

mTOR signaling regulates multiple cellular processes including cell growth, proliferation, metabolism, and survival. Mitochondria dysfunction induced by PISD deficiency was associated with reduced mTOR signaling, cap-dependent translation, and cell proliferation, indicating that it may be an effective target to slow HCC progression through its modulation of the mTOR signaling pathway. How PISD deficiency modified mTOR is not clear. Metabolomics revealed that the abundance of most cellular amino acids, except for valine, were in similar abundance between shSCR and shPISD treated cells. However, PISD treated cells had reduced abundance of various tripeptides, tetrapeptides, and pentapeptides, suggesting that sensing of these molecules by the mTOR pathway may be responsible for the reduction in cell proliferation.

In conclusion, this work provides evidence that targeting mitochondrial PE synthesis is sufficient to disrupt mitochondrial metabolism and promote oxidative stress, effects that are associated with reduced DNA synthesis and cell proliferation. Thus, targeting mitochondrial PE synthesis may be a metabolic vulnerability that can be targeted to slow HCC progression.

## Material and Methods

### Animal experiments

This study was approved by the Pennington Biomedical Research Center IACUC. Starting at two weeks of age, male and female C57BL/6J mice (Strain 000664, Jackson Labs, Bar Harbor, ME, USA) were I.P. (intraperitoneal) injected with the carcinogen diethylnitrosamine (DEN, D0516, TCI America, Montgomeryville, PA, USA) once a week for eight weeks (20 mg DEN per kg of bodyweight week one, 30 mg DEN per kg of bodyweight week two, and 50 mg DEN per kg of bodyweight weeks three through eight). All mice were weaned from their mothers at three weeks of age and received a Western diet (TD.88137, Inotiv, West Lafayette, IN, USA, 15.2% protein, 42.7% carbohydrate (with 34% sucrose), and 42% fat, by calories) which is known to accelerate liver cancer progression (47, 48). After injecting DEN once per week for eight weeks, there was a one-week washout, and then to further promote HCC the mice received thioacetamide (TAA, 172502, Millipore Sigma, Saint Louis, MO, USA) for four weeks in their drinking water (300 mg per liter of water). Control male and female mice received a purified low-fat diet (TD.05230, Inotiv, 18.7% protein, 68.7% carbohydrate, and 12.6% fat, by calories) starting when they were weaned. All the animals were group housed at 21–23°C with a 12-hour light and 12-hour dark cycle for the entire study. Mice were euthanized after a 4 hour fast. The mice received a cocktail of ketamine (10004027, Zoetis Inc, Kalamazoo, MI, USA, 120 mg of ketamine per kg of bodyweight), xylazine (061035, Covetrus North America, Dublin, OH, USA, 9 mg of xylazine per kg of bodyweight), and acepromazine (003845, Covetrus North America, 2 mg of acepromazine per kg of bodyweight) to induce anesthesia, after which a cardiac stick was performed to collect blood. Following the blood collection, the mice were cervically dislocated, and tissues were harvested. Blood samples were stored on ice until spinning at 5 000 g for 10 min at 4° C to separate cells from serum. The serum was aliquoted into a new tube and stored at −80° C until analysis.

### Tissue fixation and hematoxylin and eosin (H & E) staining

Liver sections were fixed overnight in 10% formalin (HT501128, Millipore Sigma) and then transferred to 70% ethanol the following morning. The samples were stored in 70% ethanol until embedding, sectioning, and staining. Liver slides were then stained for H & E in the Molecular Mechanisms Core at Pennington Biomedical Research Center.

### Serum alanine aminotransferase (ALT) and aspartate aminotransferase (AST) activity

Serum ALT activity was measured using a commercially available assay kit (E-BC-K235-M, MSE Supplies LLC, Tucson, AZ, USA). Serum AST activity was measured using a commercially available fluorometric assay kit (E-BC-F043, MSE Supplies LLC).

### Ki-67 staining

Immunofluorescence staining of liver sections was performed in the Molecular Mechanisms Core at Pennington Biomedical Research Center. Briefly, slides were baked for 30 min at 60° C before being run on the Leica Bond RXm using a Research Detection Kit. After standard bake and dewax on the Bond, slides were blocked for 15 min with casein blocking solution (37583, ThermoFisher Scientific) before incubating for 1 hour at 37° C with the primary antibody at a concentration of 1:300 (ab15580, abcam). After washing, the slides were incubated with a goat anti-rabbit Alexa Fluor 647 secondary antibody A32733, Invitrogen, Waltham, MA, USA) at a 1:300 dilution for 120 min. The slides were counterstained with Hoechst, coverslipped with Vector Vectashield Vibrance and scanned using a Zeiss Axioscan 7 slide scanner.

### Mitochondrial isolation

Non-tumor liver tissue and liver tumors were dissected from the same mouse and immediately placed in separate ice-cold homogenization tubes containing mitochondrial isolation media (300 mM sucrose, 10 mM HEPES, 1 mM EGTA, and 1 mg/ml BSA). The tissues were gently homogenized using a Teflon pestle, and the homogenized tissue was spun at 800 g for 10 min at 4°C. The supernatant containing mitochondria was transferred to a new tube and spun at 12 000 g for 10 min at 4°C to pellet mitochondria. After removing the supernatant, the mitochondria were resuspended in mitochondrial isolation media and then spun again at 12 000 g for 10 min at 4°C to pellet mitochondria. The supernatant was removed, and the mitochondrial pellet was immediately frozen in liquid nitrogen.

### Quantitative lipidomics

The quantitative lipidomics analysis of non-tumor and tumor mitochondria was performed by MetwareBio (Woburn, MA, USA) using ultra-performance liquid chromatography (LC) (Nexera LC-40, Shimadzu, Kyoto, Japan) tandem mass spectrometry (MS) (SCIEX, Triple Quad 6500+, Danaher Corporation, Framingham, MA, USA). Lipids were extracted by adding extraction solvent (Methyl tert-butyl ether: Methanol, 3:1, v/v) containing internal standards to each sample. After mixing samples for 15 min, ultrapure water was added, and the sample was vortexed for 1 min prior to undergoing centrifugation for 10 min at 12 000 rpm. After centrifugation, the upper organic layer was collected and evaporated using a vacuum concentrator. The dry extract was dissolved (Acetonitrile:Isopropyl Alcohol, 1:1, v/v) prior to LC-MS/MS analysis.

### Cell lines

HEPA1-6 cells (CRL-1830, ATCC, Manassas, VA, USA) were maintained in high-glucose Dulbecco’s modified Eagle’s medium (10313021, Thermo Fisher Scientific) with the addition of 10% fetal bovine serum (FBS, A5256701, Thermo Fisher Scientific) and 1% penicillin / streptomycin (15140122, Thermo Fisher Scientific). The human embryonic kidney cell line HEK293T/17 cell line (ACS-4500, ATCC) was maintained in high-glucose Dulbecco’s modified Eagle’s medium (11995065, Thermo Fisher Scientific) with addition of 10% FBS and 1% penicillin / streptomycin.

### Lentivirus production

PISD expression was inhibited using a 2nd generation lentivirus system, as previously described by our lab (49). Briefly, 3 μg of lentiviral packing plasmids psPAX2 (plasmid #12260, Addgene, Watertown, MA, USA), 1 μg of envelope expressing plasmid pMD2.G (plasmid #12259, Addgene), and 3 μg of either scrambled shRNA (plasmid #1864, Addgene) or shPISD (TRCN0000115415, Millipore Sigma) were transfected into HEK293T/17 cells to produce the lentivirus over 48 h. After 48 h the media containing the virus was collected and used to infect HEPA1-6 cells. Polybrene was added to improve transfection efficiency, and after 48 h in viral media the HEPA1-6 cells underwent puromycin selection for 72 h prior to experiments.

### RNA isolation and quantitative PCR

RNA isolation and quantitative PCR were performed as previously described by our lab (49–52). The primer sequences used included PISD/F 5’- TCTGGGGACCTTACAGAAATTGC – 3’, PISD/R 5’- GGCACAGATTTATACAGGGACAC – 3’, CEPT1/F 5’- ATGAGTGGGCATCGGTCAAC – 3’, CEPT1/R 5’- GTGGTGTCGGTAACTGAAACAA – 3’, CHKA/F 5’- GGGTGGTCTCAGTAACATGCT – 3’, CHKA/R 5’- GAACCCTGGACTCACCATCTT – 3’, PTDSS1/F 5’- GCAGGACTCTGAGCAAGGATG – 3’, PTDSS1/R 5’- GGCGAAGTACATGAGGCTGAT – 3’, PCYT2/F 5’- CGATGGCTGCTATGACATGGT – 3’, PCYT2/R 5’- GCCCCTTATGCTTGGCAATCT – 3’, TAZ/F 5’- CCCCCGCTTTGGACAGAAAAT – 3’, TAZ/R 5’- AGGCTGGAAATGATTGTGGAG – 3’.

### PISD activity assay

PISD activity in cell homogenates was measured using a fluorescence assay as previously described (53).

### Lipid extraction and thin layer chromatography

Lipids were extracted by mixing samples with a 70:30 (v:v) chloroform:methanol solution with 0.05% BHT added. Next, samples were vortexed for ~ 45–60 seconds, 2.3 ml of 0.88% KCl was added, and the mix was centrifuged at 800 g for 10 min at room temperature. The bottom layer of the sample was aspirated into a new tube and then dried using nitrogen gas. The dried lipids were suspended in chloroform and dried using nitrogen gas. This step was repeated twice. After drying for a second time, the dried lipids were suspended in chloroform before being loaded on a TLC plate (10x10 or 20x10 cm, silica gel) and allowed to dry. PE, CL, PC, PI, and PS standards were added on each plate so that the lipids in each sample could be accurately identified. The plates were developed using either a chloroform:glacial acetic acid:methanol:water (65:35:5:2) mobile phase or a chloroform:glacial acetic acid:methanol:water (85:25:5:2) mobile phase so that all phospholipids could be better visualized. The developed plates were dried prior to being sprayed with charring solution (4% phosphoric acid/5% copper sulfate) and then allowed to dry further for ~ 2 min. The plates were then heated at 190°C for ~ 15 min and the intensity of the charred lipid spots was measured using an Odyssey Infrared Imager. The intensity of the spots was quantified using ImageJ software.

### Seahorse assays

Oxygen consumption rates (OCR) and extracellular acidification rates (ECAR) were measured with a Seahorse Flux Analyzer XFe96 (Seahorse Bioscience, Billerica, MA, USA) using mitochondrial or glycolysis stress test kits, respectively, as previously described (49). After the lentiviral protocol (described above), approximately 20 000 cells were plated in the Seahorse XFe96 plate. Approximately 24 h after plating the cells the media was removed and either the mitochondrial stress test (Assay Medium: 5 mM glucose, 2 mM pyruvate and 2 mM glutamine) or glycolysis stress test (XF Assay Medium Modified DMEM (pH = 7.4) was added to the plate for ~ 1 hour prior to performing each test. After each test, the protein concentration of each well was measured using a BCA protein kit and the data was normalized to the total protein amount of the well.

### Western blotting

Samples were lysed using RIPA buffer (89900, Thermo Fisher Scientific) containing phosphatase and protease inhibitors. Total protein was measured using a Pierce^™^ BCA Protein Assay Kit (23225, Thermo Fisher Scientific). Between 12–20 μg of protein was resolved by SDS-PAGE (Bio-Rad Laboratories, Hercules, CA, USA), transferred onto nitrocellulose membrane or methanol-activated polyvinylidene fluoride (PVDF), normalized using Ponceau S staining solution (40000279, Thermo Fisher Scientific), cut to appropriate size, and blocked in 4% non-fat milk for 75 min. Blots were incubated overnight at 4°C with total OXPHOS rodent WB antibody cocktail (ab110413, Abcam), voltage-dependent anion channel (VDAC, 4866, Cell Signaling Technology, Danvers, MA, USA), peroxin-5 (PEX5, 83020, Cell Signaling Technology), glucose transporter 1 (Glut 1, 12939, Cell Signaling Technology), transketolase (TKT, 64414, Cell Signaling Technology), phosphoenolpyruvate carboxykinase 1 (PCK1, 12940, Cell Signaling Technology), enolase 1 (ENO1, 3810, Cell Signaling Technology), aldolase a (ALDOA, 8060, Cell Signaling Technology), glyceraldehyde-3-phosphate dehydrogenase (GAPDH, 2118S, Cell Signaling Technology), phosphoglycerate mutase (PGAM1, Cell Signaling Technology), superoxide dismutase 2 (SOD2, 13194, Cell Signaling Technology), dynamin-related protein 1 (DRP1, 8570, Cell Signaling Technology), mitochondrial fission factor (MFF, 84580, Cell Signaling Technology), optic atrophy 1 (OPA1, 80471, Cell Signaling Technology), mitofusin 1 (MFN1, 14739, Cell Signaling Technology), mitofusin 2 (MFN2, 9482, Cell Signaling Technology), Parkin (4211, Cell Signaling Technology), liver kinase B1 (LKB1, 3047, Cell Signaling Technology), phosphorylated LKB1^S428^ (3482, Cell Signaling Technology), adenosine monophosphate activated protein kinase (AMPK, 2532, Cell Signaling Technology), phosphorylated AMPK (2535, Cell Signaling Technology), mammalian target of rapamycin (mTOR, 2983, Cell Signaling Technology), phosphorylated mTOR^S2448^ (2971, Cell Signaling Technology), eukaryotic translation initiation factor 4E binding protein 1 (4E-BP1, 9452, Cell Signaling Technology), or phosphorylated 4E-BP1^S65^ (9451, Cell Signaling Technology). Blots were washed and incubated in the appropriate secondary antibody for 1 hour. Blots were imaged with Odyssey Li-Cor (LI-COR Biosciences, Lincoln, NE, USA) and the image analysis and densitometry quantification were performed using Li-Cor Image Studio.

### Oleate and lignoceric acid oxidation

1-^14^C oleate was purchased from PerkinElmer (NEC317050UC, Shelton, CT, USA). Fatty acid oxidation was measured using a protocol previously described (54). Briefly, cells were exposed to 0.2 μCi/ml 1-^14^C oleate for 3 h. The media was high-glucose Dulbecco’s modified Eagle’s medium (10313021, Thermo Fisher Scientific) with 10% fetal bovine serum (FBS, A5256701, Thermo Fisher Scientific) and 1% penicillin / streptomycin (15140122, Thermo Fisher Scientific). After the incubation, the media was collected and perchloric acid was added to release ^14^CO_2_ which was subsequently trapped in filter paper containing NaOH. The radioactivity of the filter paper was measured using a scintillation counter as a measure of complete fatty acid oxidation. The fatty acid oxidation data was then normalized to the total protein amount of each well.

1-^14^C Lignoceric Acid was purchased from American Radiolabeled Chemicals (ARC-0805-50, Saint Louis, MO, USA). Lignoceric acid oxidation was measured using a protocol adapted from elsewhere (55–57). Briefly, 1-^14^C lignoceric acid was dissolved in alpha-cyclodextrin prior to being added to high-glucose Dulbecco’s modified Eagle’s medium (10313021, Thermo Fisher Scientific) with 10% fetal bovine serum (FBS, A5256701, Thermo Fisher Scientific) and 1% penicillin / streptomycin (15140122, Thermo Fisher Scientific). The cells were then incubated for 5 h with 0.625 μCi/ml of 1-^14^C lignoceric acid. After the incubation, the media was collected and perchloric acid was added to release ^14^CO_2_ which was subsequently trapped in filter paper containing NaOH. The radioactivity of the filter paper was measured using a scintillation counter as a measure of complete lignoceric acid oxidation. The oxidation data was then normalized to the total protein amount of each well.

### Untargeted metabolomics

The untargeted metabolomics analysis was performed by MetwareBio using ultraperformance liquid chromatography-tandem mass spectrometry (LC-MS). Briefly, cell pellets were thawed on ice and a 500 μl solution (methanol:water = 4:1, V/V) containing internal standard was mixed with the cell sample and vortexed for 3 min. The sample was placed in liquid nitrogen for 5 min, on the dry ice for 5 min, and then thawed on ice and vortexed for 2 min. This freeze-thaw cycle was repeated three times in total. The sample was centrifuged at 12 000 rpm for 10 min at 4° C. Next, 300 μl of the supernatant was collected and placed in −20° C for 30 min. The sample was centrifuged again at 12 000 rpm for 3 min at 4° C. A 200 μl aliquot of the supernatant was used for LC-MS analysis. The metabolite abundance was normalized to total protein for each sample.

### Media glucose and lactate concentrations

The rate of glucose disappearance or lactate appearance in the media was measured over a 24-hour period. The media used was high-glucose Dulbecco’s modified Eagle’s medium (10313021, Thermo Fisher Scientific) with 10% fetal bovine serum (FBS, A5256701, Thermo Fisher Scientific) and 1% penicillin / streptomycin (15140122, Thermo Fisher Scientific). Briefly, the media of cells was refreshed, and an aliquot of fresh media was stored at −80° C. After 24 h, an aliquot of the used media incubating with the cells was collected and stored at −80° C. The glucose concentration of the fresh and used media was measured using a commercially available colorimetric kit (937–03001, FUJIFILM Healthcare Americas Corporation, Lexington, MA, USA). The lactate concentration of the fresh and used media was measured using a commercially available colorimetric kit (NBP3-25788, Novus Biologicals LLC, Centennial, CO, USA). The difference in glucose or lactate concentration between the fresh and used media was calculated to determine the change in concentration over the 24-hour incubation period. The glucose or lactate concentration was normalized to total protein content of the well to correct for any differences in cell number.

### Glucose uptake and oxidation

1-^14^C Glucose (NEC043X050UC), 6-^14^C glucose (NEC045X050UC), and 1-^14^C 2-deoxyglucose (NEC495A050UC) were purchased from PerkinElmer.

For experiments involving 1-^14^C or 6-^14^C glucose, the cells were serum starved for 3 h before experiments. The serum starve media was high-glucose Dulbecco’s modified Eagle’s medium (10313021, Thermo Fisher Scientific) with 1% penicillin / streptomycin (15140122, Thermo Fisher Scientific). After serum starvation, cells were exposed to 0.2 μCi/ml of 1-^14^C or 6-^14^C glucose for 3 h in serum free media. After the incubation, the media was collected and perchloric acid was added to release ^14^CO_2_ which was subsequently trapped in filter paper containing NaOH. The radioactivity of the filter paper was measured using a scintillation counter. The oxidation data was normalized to the total protein content of each well.

For experiments involving 1-^14^C 2-deoxy-glucose the cells were serum starved for 3 h prior to experiments. After serum starvation, 0.5 μCi/ml of tracer was added to cells for 20 min. Following the incubation, the cells were washed twice with ice cold phosphate-buffered saline (10010023, Thermo Fisher Scientific) and then RIPA buffer (89900, Thermo Fisher Scientific) was added to lyse the cells, and the radioactivity of the cell lysate was measured using a scintillation counter.

### Fluorescent imaging of glucose uptake

A commercially available kit and glucose probe (UP03-10, Dojindo Molecular Technologies, Inc, Rockville, MD, USA) were used to measure glucose uptake with an ECHO Revolve fluorescent microscope (ECHO: A BICO Company, San Diego, CA, USA). Nucblue (R37605, Thermo Fisher Scientific) was used to stain nuclei during these glucose uptake experiments. Briefly, the glucose probe and NucBlue were added to glucose and fetal bovine serum free Dulbecco’s Modified Eagle Medium (A1443001, ThermoFisher Scientific). Next, the media on the cells was suctioned off and the cells were washed once with the glucose and fetal bovine serum free media. Next, the media containing the glucose probe and NucBlue were added to the cells for 10 min at 37°C. After 10 min, the cells were washed twice with PBS, glucose and serum free media without the glucose probe and NucBlue was added back to the wells, and the plates were imaged.

### Mitochondrial reactive oxygen species (ROS) determination

The MitoSOX^™^ (M36008, Thermo Fisher Scientific) red mitochondrial dye was used to measure mitochondria superoxide production. Cells were treated with 500 nM MitoSOX^™^ red reagent, which was added to high-glucose Dulbecco’s modified Eagle’s medium (10313021, Thermo Fisher Scientific) with 1% penicillin / streptomycin (15140122, Thermo Fisher Scientific) for 20 min at 37°C. Cells were washed three times in PBS, stained in NucBlue for 10 min at 37°C, washed three times in PBS, and then imaged in phenol free media with an ECHO Revolve fluorescence microscope.

### Mitophagy assay and extracellular mitochondria

Mitophagy was assessed by measuring the colocalization of MitoTracker Green (M7514, ThermoFisher Scientific) with LysoTracker Red (L7528, ThermoFisher Scientific) using an ECHO Revolve fluorescent microscope. ImageJ software was used to quantify the Manders Colocalization Coefficient. Briefly, the dyes were added to high-glucose Dulbecco’s modified Eagle’s medium (10313021, Thermo Fisher Scientific) with 1% penicillin / streptomycin (15140122, Thermo Fisher Scientific). Next, the media containing dyes was added to the cells for 30 min at 37°C. After 30 min, the cells were washed twice with PBS and then media without dyes was added back to each well, and the cells were imaged. Extracellular mitochondria were also assessed with MitoTracker Green in these same images.

### Cell proliferation

DNA synthesis (S phase of cell cycle) was measured using a Click-iT^®^ EdU Alexa Fluor^®^ 488 Imaging Kit (C10337, Thermo Fisher Scientific). Cells were grown in DMEM and plated on an 8 chamber ibidi slide (ibidi USA, Inc., Fitchburg, WI, USA). After 24 h the cells were labeled with EdU (5-ethynyl-2’-deoxyuridine) by incubating them with 10 μM of EdU at 37°C for one hour. After the incubation, the cells were washed and then subsequently fixed in formaldehyde-PBS solution for 15 min and then permeabilized in Triton X-100 PBS solution for 20 min, both of which were performed at room temperature. The Click-iT reaction cocktail was then added to the cells, after which a Hoechst-PBS solution was used to stain Nuclei prior to imaging on an ECHO Revolve fluorescence microscope. Images were analyzed using CellProfiler cell image analysis software.

A Cell Counting Kit (WST-8 / CCK8) (ab228554, abcam, Boston, MA, USA) was used to measure cell proliferation. Briefly, 10,000 shSCR or shPISD treated cells were plated in a 96-well clear flat bottom plate and incubated for 24 h in a 37°C, 5% CO_2_ incubator. We followed the manufacturer’s instructions using a total volume of 100 μl/well of medium with cells and two blank wells (medium without cells). The first measure (baseline) was made 24 h after seeding the cells on the 96-well plate. We changed the medium to a fresh one containing 10 μl/well of WST-8 solution, followed by one hour of incubation. The colored product was quantitatively measured at 460 nm using a microplate reader (Bio-Rad Laboratories). After measuring the baseline absorbance, the media was refreshed every 24 h until a second measure was made 72 h later, using the same procedure described above. Cell proliferation is directly proportional to the absorbance measured in the media.

## Statistical analysis

GraphPad Prism version 10.0.2 was used to analyze the data and generate the figures. The data are presented as means ± S.E.M.. Where appropriate either t-tests or two-way ANOVAs were used for statistical analysis. For two-way ANOVA’s, follow-up Bonferroni multiple comparison tests were used to identify specific differences. The BioRender app (www.biorender.com) was used to generate some images. ImageJ was used to determine the fluorescent intensity of the images. Bioinformatics analysis of the lipidomics and metabolomics data was performed by MetwareBio.

## Supplementary Material

This is a list of supplementary files associated with this preprint. Click to download.

- SupplementalTable1.MitochondrialLipidomics.xlsx
- SupplementalTable2.Metabolomics.xlsx

## Data Availability

The datasets generated during and/or analyzed during the current study are available from the corresponding author on reasonable request.
